# Description and comparison of national surveillance systems and response measures for *Aedes*-borne diseases in France, Italy and Portugal: a benchmarking study, 2023

**DOI:** 10.2807/1560-7917.ES.2025.30.15.2400515

**Published:** 2025-04-17

**Authors:** Emmanouil Alexandros Fotakis, Berta Grau-Pujol, David Kelly, Pedro Pinto Leite, João Vieira Martins, Maria João Alves, Marco Di Luca, Giulietta Venturi, Federica Ferraro, Florian Franke, Clément Pietin, Clémentine Calba, Tanja Charles, Flavia Riccardo, Paula Vasconcelos, Lauriane Ramalli

**Affiliations:** 1 ECDC Fellowship Programme, Field Epidemiology path (EPIET), European Centre for Disease Prevention and Control (ECDC), Stockholm, Sweden; 2 Department of Infectious Diseases, Istituto Superiore di Sanità, Rome, Italy; 3 Directorate of Information and Analysis, Directorate-General of Health, Lisbon, Portugal; 4 Public Health Emergency Operations Centre, Directorate-General of Health, Lisbon, Portugal; 5 Santé Publique France, French National Public Health Agency, Marseille, France; 6 Centre for Vectors and Infectious Diseases Research, National Institute of Health Doutor Ricardo Jorge, Lisbon, Portugal; 7 Ministry of Health, Rome, Italy; 8 Department of Public Health and Environment, Agence Régionale de Santé, Provence Alpes-Côte d’Azur, France; 9 Robert Koch Institute, Department for Infectious Disease Epidemiology Unit for crisis management, outbreak investigations and training programmes, Berlin, Germany

**Keywords:** Cross-border, preparedness, arboviruses, emerging infectious disease, *Aedes*-borne disease, surveillance

## Abstract

**Background:**

Regions of southern Europe are increasingly colonised by *Aedes albopictus,* with incidence of autochthonous dengue cases rising in recent years.

**Aim:**

We describe and compare *Aedes*-borne disease (dengue, chikungunya and Zika) incidence from 2017 to 2023, and the surveillance systems and response measures operating in France, Italy and Portugal in 2023, to improve surveillance, prevention, preparedness and response in Europe.

**Methods:**

We performed a benchmarking analysis to systematically capture the systems used in each country. We collected data from key-informant interviews, national guidelines, reports and scientific literature using a standardised questionnaire adapted from the European Centre for Disease Prevention and Control framework.

**Results:**

All three countries have an integrated surveillance system for *Aedes*-borne diseases and share similarities in surveillance type, geographic coverage and case definitions. Differences entail mainly event-based and active surveillance activities. Geographic coverage of vector surveillance is national in France and Portugal but regional in Italy. In response to autochthonous transmission, all countries implement/foresee active case-finding and blood safety protocols, while France and Italy strongly rely on vector control. Upon vector detection in non-colonised areas, the three countries implement ad hoc entomological surveillance and vector control.

**Conclusions:**

Surveillance systems and response measures in France, Italy and Portugal are broadly similar, with variations reflecting differences in healthcare system organisation (centralised in Portugal and France, regionalised in Italy), *Ae. albopictus* distribution and local transmission of *Aedes-*borne diseases. Risk-based surveillance, considering the national and cross-border epidemiological and entomological situations, can strengthen preparedness and early warning for *Aedes*-borne diseases in Europe.

**Key public health message**

**What did you want to address in this study and why?**

Different human and vector surveillance systems operate nationally to detect and report cases of *Aedes*-borne disease and *Aedes* vector presence and population dynamics in southern Europe. We aimed to describe and compare surveillance systems and response measures to *Aedes*-borne disease in France, Italy and Portugal to improve preparedness and response to emerging arbovirus diseases in Europe.

**What have we learnt from this study?**

France, Italy and Portugal have integrated surveillance systems for *Aedes*-borne disease that share similarities in their coverage, surveillance type, disease notification and case definitions. France also operates active surveillance and Italy and Portugal event-based surveillance. The three countries implement invasive *Aedes* spp. surveillance in colonised areas and at points of entry. Regional variation exists due to differences in vector colonisation and health system organisation. **What are the implications of your findings for public health?**

Cross-border collaboration between neighbouring European countries can facilitate shared good practices in surveillance and response to *Aedes*-borne diseases strengthening preparedness and early warning for these diseases in Europe.

## Introduction

*Aedes* mosquitoes, known vectors for several arboviruses, are increasingly colonising southern Europe. *Aedes albopictus* expanded its range from 8 to 13 countries within the European Union/European Economic Area (EU/EEA) between 2013 and 2023 [1], while *Ae. aegypti* has an established presence along the coasts of the Black Sea and was recently introduced in Cyprus. Moreover, risk mapping indicates further expansion of *Aedes* vectors in the Mediterranean region and potential incursion into northern Europe as a result of climate change and mobility of goods and people [2].

Colonisation of parts of Europe by *Ae. albopictus* and *Ae. aegypti* raises the local transmission risk of arboviral diseases, particularly dengue, Zika and chikungunya [3]. These diseases are associated with serious clinical presentations and outcomes, as well as asymptomatic infection. While dengue, Zika and chikungunya have distinctive clinical features, they are not easily distinguishable in the initial phase of primary vector-transmitted infection and no aetiological treatments are available [3,4]. Vaccines are currently available for dengue and chikungunya but require further study to determine their use in both endemic and non-endemic countries.

Despite efforts to control *Aedes*-borne diseases, local transmission events to humans have occurred, both in continental Europe and in sub-tropical European territories. Following a first outbreak in 2007 [5], Italy reported 499 probable and confirmed autochthonous cases of chikungunya in 2017. France reported its first autochthonous dengue cases in mainland France in 2010, with multiple episodes since [6]. Portugal reported its first dengue outbreak in 2012 on Madeira Island (situated in the North Atlantic Ocean), with 1,080 confirmed autochthonous dengue cases.

In response to the evolving arboviral epidemiological situation in Europe, there is a pressing need to comprehensively overview *Aedes*-borne diseases and vector surveillance and response in southern Europe in the interests of strengthening prevention, preparedness and control measures in the EU/EEA. This involves describing and comparing existing systems, identifying each system’s strengths and weaknesses and pinpointing good practices within epidemiological and entomological contexts. Through a surveillance system benchmarking analysis approach [7], with special focus on France, Italy and Portugal, we aim to provide insights and recommendations for enhancing dengue, Zika, chikungunya and *Aedes* spp. surveillance, prevention, preparedness and response at national and international level.

## Methods

### Epidemiological and entomological data

We obtained national and/or regional surveillance data from France (excluding overseas territories), Italy and Portugal (including Madeira and the Azores) to provide an epidemiological overview of the detection and reporting of dengue, Zika and chikungunya cases in each country. For each country, we obtained autochthonous and imported human cases (number and incidence rates per 1,000,000 population), number of autochthonous transmission clusters and regions affected, and the presence of invasive *Aedes* mosquito species for the period 2017–2023. We calculated the annual incidence of autochthonous cases per 1,000,000 population, and the percentage of total geographical zones (nomenclature of territorial units for statistics level 3 (NUTS 3) for Italy and France and local administrative units level 1 (LAU1) for Portugal) colonised by the *Aedes* spp vector.

### Benchmarking analysis

Using a benchmarking analysis approach described in [7], we systematically described and compared the human surveillance systems used for dengue, Zika and chikungunya surveillance in France (excluding overseas territories), Italy and Portugal (including Madeira and the Azores) in 2023, as well as each country’s entomological surveillance system targeting invasive *Aedes* mosquitoes.

The benchmarking objects and criteria we used were based on the comprehensive set of surveillance system descriptor elements listed in the European Centre for Disease Prevention and Control (ECDC) technical document ‘Data quality monitoring and surveillance system evaluation’ [8]. We adapted and tailored this list to the context and aims of the current study and considered new surveillance and response elements for inclusion in our benchmark questionnaire and reporting framework [8]. We defined the selected benchmarking objects and criteria through a consensus decision making process led by ECDC Fellowship programme field epidemiology path (EPIET) fellows ensuring standardised information coding. We selected 13 benchmarking objects and 59 criteria (Table 1). The descriptions of all criteria are provided in Supplementary Table S1.

**Table 1:** Benchmarking objects and criteria used for the description of the human surveillance systems for dengue, Zika and chikungunya and for the surveillance systems for *Aedes* spp. mosquitoes in place in France, Italy and Portugal, 2023

	Benchmark objects	Benchmarking criteria
Human surveillance system	1. Surveillance objectives	Defined within national and/or regional surveillance plans
	2. Case definitions	The use of ECDC definitions; classification system; case criteria
	3. National surveillance data flow	The stakeholders in case reporting; data flow; feedback mechanisms
	4. Population under surveillance	The target population; risk groups
	5. Geographic coverage	The national coverage; number of regions covered; regional variations
	6. Type of surveillance	Passive; active; comprehensive; sentinel; syndromic-based, event-based; indicator-based; risk-based; mandatory vs voluntary, seasonality; citizen surveillance; early warning components
	7. Legal framework	The legislation mandating surveillance e.g. notifiable disease status
	8. Specification of information	Data variables; individual vs aggregated data; collection frequency
	9. Reporting format	The mode and tools for data reporting
	10. Data entry	Interfaces, processes and software specification
	11. Database architecture	Administrative level of database management; server location; data linkage
	12. Alert threshold for response	The established threshold for triggering response and outbreak definitions
	13. Response actions	Epidemiological investigations; case finding; testing strategies; blood and organ donation safety measures; ad hoc entomological surveillance; communication of alerts
Surveillance systems for *Aedes* spp.	1. Surveillance objectives	Defined within national and/or regional surveillance plans
	2. Surveillance data flow	The stakeholders in entomological reporting; data flow; feedback mechanisms
	3. Geographic coverage	The national coverage; number of regions covered; regional variations; target areas
	4. Type of surveillance	Passive; active; systematic; participative; mandatory vs voluntary; risk based; seasonality; event based
	5. Specification of information	Presence/absence; abundance and seasonal dynamics; molecular xenomonitoring^a^; insecticide resistance; specimen collection and data reporting frequency
	6. Reporting format	The mode and tools for data reporting
	7. Data entry	Interfaces, processes and software specification
	8. Alert threshold for response	The established entomological threshold for triggering response
	9. Response actions	Vector control; molecular xenomonitoring^a^; ad hoc entomological surveillance; national/international communication

ECDC: European Centre for Disease Prevention and Control.

^a^ DNA/RNA detection of human pathogens in arthropod vectors.

We followed a similar process for the entomological surveillance system targeting *Aedes* mosquitoes in each of the three countries, including response measures to routine entomological surveillance findings. Key documents used for this purpose included the ECDC technical report ‘Guidelines for the surveillance of invasive mosquitoes in Europe’ [9], and the World Health Organization (WHO) framework for national surveillance and control plans for *Aedes* vector [10]. We selected a total of 9 objects and 39 criteria for the analysis (Table 1). The descriptions of all criteria are provided in Supplementary Table S2.

### Data and information collection

We created a standardised Excel benchmark tool to collect information from each of the three countries. We reviewed the latest guidelines, national and regional surveillance plans, surveillance reports and scientific literature, extracting all relevant data that matched our benchmark criteria between January and March 2024. Moreover, we conducted key informant interviews with surveillance system focal experts from the national and regional public health institutions of each country: Santé Publique France (France), Istituto Superiore di Sanità (ISS) (Italy) and Direção-Geral da Saúde (Portugal), as well as with other national partners. Using the benchmark tool in a questionnaire framework allowed for flexibility in the conversation while ensuring all key topics in our tool were covered. We summarised the findings and reviewed the preliminary data collected by authors EAF, BGP and DK to further ensure homogenous coding among the three countries.

### Identification of similarities, differences, strengths and weaknesses

The data and information from each country were critically assessed and compared descriptively (integrating several quantitative indicators described in Supplementary Table S1) to guide recommendations to improve the surveillance systems for prevention, preparedness and control against dengue, Zika and chikungunya transmission at national and international level. Similarities and differences were highlighted, and emphasis was given to identifying good practices and weaknesses, while taking into account each country’s epidemiological and entomological context.

## Results

### Epidemiological and entomological situation in France, Italy and Portugal

For the period 2017 to 2023, an increasing number of confirmed autochthonous dengue clusters and cases were reported in mainland France (peak incidence of 1/1,000,000 population in 2022) and Italy (peak incidence of 1.4/1,000,000 in 2023) (Table 2). No autochthonous cases were reported during this period in mainland Portugal. Chikungunya clusters were reported in both France and Italy in 2017, while locally acquired Zika cases were reported only in France in 2019.

**Table 2:** Epidemiology of *Aedes*-borne diseases (dengue, Zika and chikungunya) in France (mainland), Italy and Portugal, 2017–2023

*Aedes*-borne disease	France							Italy							Portugal						
**Dengue**	**2017**	**2018**	**2019**	**2020**	**2021**	**2022**	**2023**	**2017**	**2018**	**2019**	**2020**	**2021**	**2022**	**2023**	**2017**	**2018**	**2019**	**2020**	**2021**	**2022**	**2023**
Imported cases	137	189	657	834	164	272	2,019^c^	95	108	185	19	11	114	295	10	14	24	2	7	12	40
Autochthonous cases	0	8	9	14	2	66	42	0	0	0	11	0	0	82	0	0	0	0	0	0	0
**Autochthonous incidence per 1,000,000^a^**	0.0	0.1	0.1	0.2	0.0	1.0	0.6	0	0	0	0.2	0	0	1.4	0	0	0	0	0	0	0
Autochthonous clusters^b^	0	3	2	6	2	9	8	0	0	0	1	0	0	4	0	0	0	0	0	0	0
Regions with clusters	0	2	2	2	2	3	4	0	0	0	1	0	0	2	0	0	0	0	0	0	0
**Zika**	**2017**	**2018**	**2019**	**2020**	**2021**	**2022**	**2023**	**2017**	**2018**	**2019**	**2020**	**2021**	**2022**	**2023**	**2017**	**2018**	**2019**	**2020**	**2021**	**2022**	**2023**
Imported cases	15	0	6	1	0	3	9	25	1	4	3	0	1	9	1	0	0	1	0	1	3
Autochthonous cases	0	0	3	0	0	0	0	0	0	0	0	0	0	0	0	0	0	0	0	0	0
**Autochthonous incidence per 1,000,000^a^**	0	0	0.1	0	0	0	0	0	0	0	0	0	0	0	0	0	0	0	0	0	0
Autochthonous clusters^b^	0	0	1	0	0	0	0	0	0	0	0	0	0	0	0	0	0	0	0	0	0
Regions with clusters	0	0	1	0	0	0	0	0	0	0	0	0	0	0	0	0	0	0	0	0	0
**Chikungunya**	**2017**	**2018**	**2019**	**2020**	**2021**	**2022**	**2023**	**2017**	**2018**	**2019**	**2020**	**2021**	**2022**	**2023**	**2017**	**2018**	**2019**	**2020**	**2021**	**2022**	**2023**
Imported cases	4	6	56	6	3	22	30	12	5	18	3	0	0	7	0	1	0	0	0	0	2
Autochthonous cases	17	0	0	0	0	0	0	277	0	0	0	0	0	0	0	0	0	0	0	0	0
**Autochthonous incidence per 1,000,000^a^**	0.3	0	0	0	0	0	0	4.6	0	0	0	0	0	0	0	0	0	0	0	0	0
Autochthonous clusters^b^	2	0	0	0	0	0	0	3	0	0	0	0	0	0	0	0	0	0	0	0	0
Regions with clusters	1	0	0	0	0	0	0	2	0	0	0	0	0	0	0	0	0	0	0	0	0

^a^ Calculated using a denominator of total populations reported for each year in the Eurostat database ‘Demographic balance and crude rates’ (DEMO_PJAN) [23].

^b^ France and Italy: a cluster is defined as ≥ 1 autochthonous case with spatiotemporal proximity as defined in Dengue – Santé publique France and National surveillance system of arboviral diseases: regular bulletins - Istituto Superiore di Sanità

Portugal: a cluster is defined as ≥ 1 autochthonous case.

^c^ Elevated due to epidemics in French overseas territory. We included cases reported in mainland France (even if their origin of infection was from overseas). We do not include cases reported in the French overseas territories.

*Aedes albopictus* colonisation increased in France from 44% to 81% of mainland geographical zones (NUTS 3) and in Portugal from 3% to 6% of geographical zones (LAU1) during the period 2017 to 2023. Italy reported in excess of 99% of geographical zones (NUTS 3) colonised since 2018 (Table 3).

**Table 3:** *Aedes* species detection in France (mainland), Italy and Portugal, 2017–2023

*Aedes albopictus* vector	France							Italy							Portugal						
**Year**	**2017**	**2018**	**2019**	**2020**	**2021**	**2022**	**2023**	**2017**	**2018**	**2019**	**2020**	**2021**	**2022**	**2023**	**2017**	**2018**	**2019**	**2020**	**2021**	**2022**	**2023**
Geographical zones colonised^a^	42	49	58	61	67	71	78	109	109	109	109	109	110	110	9	10	10	11	14	15	19
Territory colonised (%)	44	51	60	64	70	74	81	99	99	99	99	99	100	100	3	3	3	4	5	5	6
**First year *Ae. albopictus* documented**	**2004**							**1990**							**2017**						
*Ae. japonicus*	2013							2015							Not detected						
*Ae. koreicus*	Not detected							2011							Not detected						
*Ae. aegypti*	Not detected							Not detected							2005^b^						

^a^ France: 96 total administrative areas (départements) (NUTS 3, Nomenclature of Territorial Units for Statistics). Source: link.

Italy: 107 total provinces (province) (NUTS 3). Source: link.

Portugal: 308 municipalities (concelhos) (LAU 1, Local Administrative Units). Source: link.

^b^ *Ae. aegypti* was detected on Madeira Island.

### Comparison of dengue, Zika and chikungunya human surveillance and response in France, Italy and Portugal

*Aedes*-borne arbovirus surveillance is regulated in France by the Health Ministerial decree updated in 2019 as the *Guide to prevent and evaluate risks of arbovirus transmission in mainland France* [11]; in Italy by the *National Plan for prevention, surveillance and response to Arboviruses 2020–2025* [12]; and in Portugal by the *Law 81/2009 on the National Public Health Surveillance System* from August 2009 [13]. All countries have an integrated surveillance system for dengue, Zika and chikungunya yet retain the three arboviruses and associated diseases as separate ontological entities across disease notification and reporting. Country-specific surveillance system descriptions provided hereafter refer to all three diseases unless specified otherwise.

**Figure 1:**
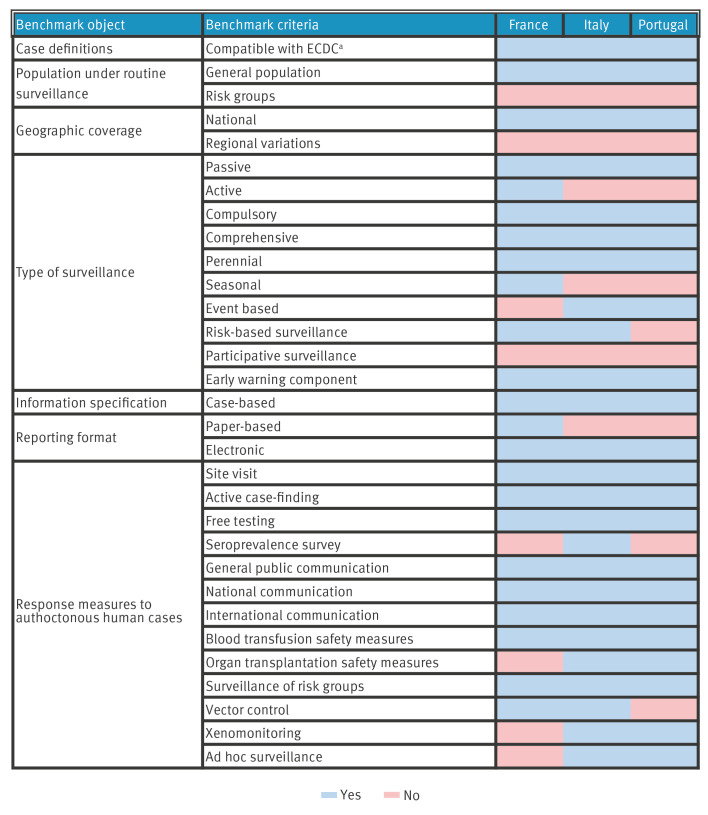
Routine human surveillance systems and response measures to *Aedes*-borne disease in France, Italy and Portugal, excluding overseas territories, 2023

The primary surveillance objective of each of the three countries is to reduce the risk of autochthonous transmission. In each country, distinct case definitions are applied for dengue, Zika and chikungunya, including disease-specific clinical, laboratory and epidemiological criteria. While these are compatible with those of ECDC, France and Italy use a three-tiered classification system (i.e. possible/probable/confirmed case) (Supplementary Table S3), incorporating slightly different clinical and/or epidemiological criteria (Figure 1).

ECDC: European Centre for Disease Prevention and Control.

^a^ Although all country case definitions are compatible with those of ECDC, slightly different clinical and/or epidemiological criteria are applied in Italy and France compared to the ECDC case definitions. For example, in Italy, clinical criteria for a probable case of classical dengue fever include fever and at least two of the following symptoms: nausea, vomiting, ocular or retro-orbital pain, headache, maculopapular skin rash, myalgia, arthralgia, compared to only fever in the ECDC case definition.

Results presented in the figure represent a national level summary of findings to facilitate cross-border comparison.

Results of all the benchmark objects, listed in Table 1, including explanatory notes, are described in Supplementary Table S3.

Surveillance data flow is similar in all three countries, see Supplementary Figure S1. Regarding event-based surveillance, Italy and Portugal produce a weekly Epidemic Intelligence (EI) bulletin including vector-borne diseases, summarising key public health alerts based on both case-based and event-based data and information management. In each country, the population under routine surveillance comprises the general population, and all surveillance systems display a national and regionally uniform geographic coverage.

France, Italy and Portugal practice passive case reporting. Surveillance is comprehensive and runs year-round. Additionally, Italy and Portugal conduct event-based surveillance through the use of EI (i.e. Early Warning and Response System (EWRS), EpiPulse, MedISys, Epidemic Intelligence from Open Source (EIOS) and social media). France employs active surveillance during the high-risk season (May–November), where retrospective checking of laboratory result databases is performed to actively search for unreported cases. Early warning functions are integrated within each of the different country systems, involving automated and real-time electronic mail case-notification alerts circulated to a predefined network of surveillance focal points within each country.

In all three countries, cases are notifiable under national law. Cases are notified and reported individually and on a daily basis across the respective surveillance data flows. Annual summary reports are shared with ECDC. Mandatory notification/reporting information is comparable among the three countries and includes case demographic data, symptomatology, type of laboratory diagnostics conducted, laboratory results, origin status and country of import and case classification.

In all three countries, the human surveillance databases are centralised at national level. In Italy, separate regional databases are also present at regional level reflecting the decentralised national health system (NHS) organisation. These databases integrate laboratory, clinical and epidemiological surveillance case-based data, and data are manually entered in the respective web-based portals. Data linkage between epidemiological and entomological datasets is available only in France, where a specific system allows the informing of vector control measures based on case detection.

Alert thresholds prompting public health response are defined as ≥ 1 probable/confirmed autochthonous cases in all three countries. Outbreaks in France and Italy are defined as a cluster of ≥ 2 autochthonous cases with spatiotemporal proximity vs a single autochthonous case in Portugal. In all three countries, automatic emails alerting the presence of probable and confirmed cases are sent in real-time upon notification to the national public health authorities, national reference laboratories (except for France) and substances of human origin (SoHO) national contact points. This leads to the activation and coordination of rapid response with regional counterparts and the required timely communication with international partner institutions. International communication is carried out through Epipulse, EWRS, personal communication and scientific publications.

In response to alerts, all three countries implement field visits, furthering epidemiological investigations, and local level active case-finding, involving household contact surveys or voluntary screening campaigns (in the case of Italy). Following a confirmed autochthonous case, all countries implement blood safety protocols. Testing and suspension of donations are locally activated, including a 28-day donor deferral upon entry in autochthonous transmission risk zones in France and Italy, extending to 4 months in Portugal. Safety protocols are extended to tissue/organ transplantations in Italy and Portugal.

France and Italy’s responses to probable/confirmed autochthonous case alerts focus on systematic vector control interventions. Vector control efforts involve applying adulticides (i.e use of pyrethroid based insecticides) and/or larvicides (i.e. *Bacillus thuringensis var. israeliensis* (Bti) or insect growth regulators (IGRs) in close proximity (radius ≤ 200 m) to designated high-risk areas. These measures are often complemented by environmental management interventions (i.e. elimination of larval breeding sites). In response to autochthonous cases, local level ad hoc entomological surveillance and molecular xenomonitoring (i.e. DNA/RNA detection of human pathogens in arthropod vectors) activities are carried out in Italy and are planned in Portugal.

### Comparison of invasive *Aedes* species surveillance and response

**Figure 2:**
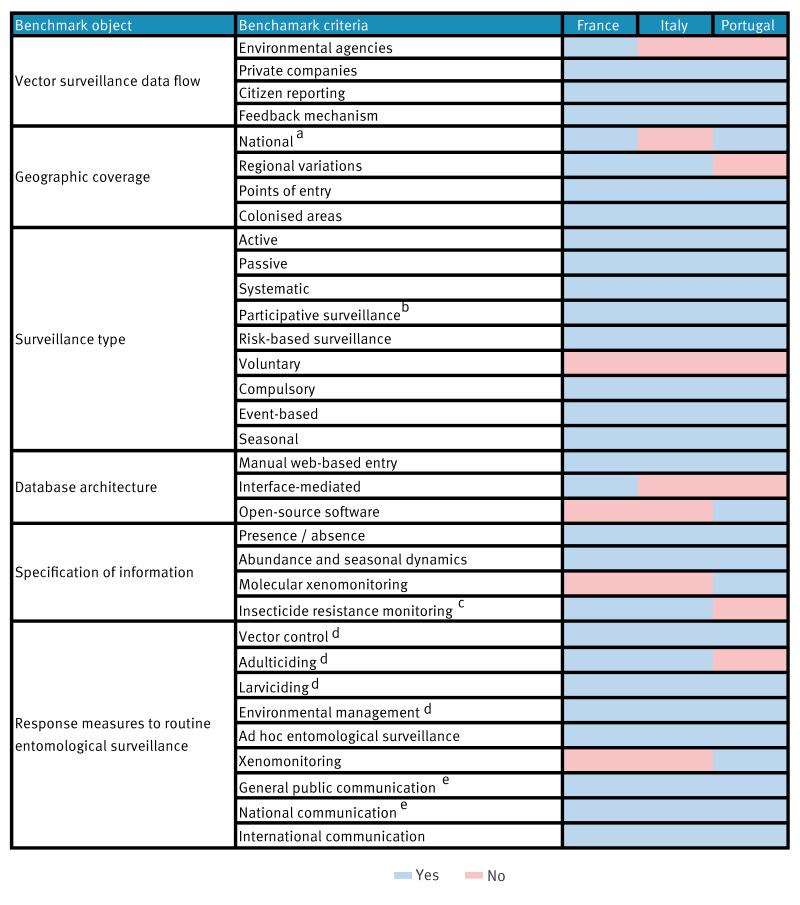
Routine vector surveillance systems and response measures to *Aedes* species in France, Italy and Portugal (excluding overseas territories) as of 2023

^a^ In Italy *Aedes* vector surveillance coverage is regional, future implementation of a national coverage system is planned.

^b^ In Italy participative surveillance is currently implemented as part of pilot/proof of concepts experiences.

^c^ Resistance monitoring is conducted periodically in France and systematically in Emilia-Romagna and Veneto regions of Italy.

^d^ Vector control measures are foreseen upon invasive *Aedes* specimen detection in previously non-colonised areas. In the absence of autochthonous transmission, vector control measures are not routinely performed in known colonised areas.

^e^ Communications include the detection of invasive species in previously non colonised areas but also vector abundance fluctuations in previously/known colonised areas.

In France and Portugal, vector surveillance data flows extend from local/regional to national level, whereas in Italy surveillance data flows are subnational with communication of any unusual/unexpected findings to the ISS and the Ministry of Health (MoH). Environmental agencies and private sector vector control operators are involved in data collection in France, while private sector vector control operators, the ISS, universities and regional Zooprophylactic Institutes (IZSs) facilitate data collection in several Italian regions (Figure 2). Feedback mechanisms are established in all countries. In France, there is feedback between the National Environmental Health Agency (ANSES), the MoH and regional health authorities, and from these to the vector control operators. In Italy, there is feedback between the MoH, ISS, IZSs, public veterinary institutes and universities involved in surveillance activities, and in Portugal feedback is between the National Institute of Health and regional-national health authorities. In all three countries, primary surveillance-target vector species include *Ae. albopictus*, *Ae. aegypti*, *Ae. japonicus* and *Ae. koreicus.*

Results presented in the figure represent a national level summary of findings to facilitate cross-border comparison.

Results of all the benchmark objects, listed in Table 2, are described in Supplementary Table S4.

*Aedes* surveillance has a national geographic coverage in France and Portugal, while in Italy coverage is regional, reflecting the regionalised organisation of its NHS (future implementation of a national coverage system is planned). Surveillance protocols are uniform across regions in Portugal. Regional variations in spatiotemporal coverage, intensity and mosquito trapping protocols exist in France and Italy.

The three countries systematically employ active surveillance (using adult and oviposition traps, complemented with larval collections), covering both colonised areas and potential points of entry (PoE) (e.g. airports and seaports). Italy mostly focuses its surveillance efforts on PoE and invasive *Aedes* species other than *Ae. albopictus*. Surveillance is seasonal in France and Italy and year-round for PoE in Portugal. Surveillance is risk-based, guided by varied indicators between countries and regions in Italy. Indicator reporting is compulsory in all countries. Active surveillance is complemented by participative surveillance through citizen reporting apps in France [14] and in Portugal [15] and currently implemented as part of pilot/proof of concepts experiences in Italy [16]. Community vector-surveillance is integrated within epidemic intelligence activities in Portugal - upon citizen-mosquito reporting, house visits are made to verify vector presence.

During the vector season, mosquito traps are deployed weekly in Portugal, weekly or biweekly (depending on trap type) in Italy and biweekly in France. Data are reported and collected electronically in national (France and Portugal) and regional (Italy) web-based portals. Frequency of data reporting is flexible. Information reported in each of the three countries includes vector species, presence/absence, vector abundance and dynamics data aggregated at the municipality level. Molecular xenomonitoring, irrespective of probable/confirmed case occurrence, is systematically implemented in Portugal. In Italy, xenomonitoring is currently performed reactively, as previously described. However, a more systematic implementation is planned for the future. France and Italy currently conduct insecticide resistance monitoring (periodically in France and systematically in the regions of Emilia-Romagna and Veneto in Italy, with the aim to extend this further across the country).

In all three countries, response measures apply to *Aedes* spp. detection in previously non-colonised areas, with less emphasis on vector abundance in already colonised areas. Specifically, upon detection of ≥ 1 invasive *Aedes* specimen in a previously non-colonised area, vector control interventions are performed in all three countries (i.e. larviciding, environmental management and adulticiding in France and Italy). Responses in all three countries include ad hoc intensified entomological surveillance activities in the affected and neighbouring areas, and xenomonitoring in Portugal. When vector activity increases in colonised areas or invasive species are found in new areas, all three countries notify the general public. Portugal issues national stakeholder reports, while all three countries reported colonisation distribution to ECDC if a new species is found.

## Discussion

The evolution of *Aedes-*borne disease surveillance and response in France, Italy and Portugal is shaped by three main drivers: (i) healthcare system organisation (centralised in France and Portugal and regionalised in Italy), (ii) extent of *Ae. albopictus* colonisation, and (iii) epidemiology of local transmission of *Aedes-*borne diseases in each country.

The surveillance of *Aedes*-borne diseases in humans is performed through similar and well-established national surveillance systems in France, Italy and Portugal. The operating systems share commonalities in coverage, surveillance type, disease notification and case definitions, reflecting similar *Aedes*-borne disease prioritisation in the three countries. Nonetheless, the surveillance systems differ in certain aspects (e.g. active human case surveillance in France and event-based surveillance in Italy and Portugal), likely indicating differing historic adaptations to increasing vector colonisation, recent history of autochthonous *Aedes*-borne disease clusters and rising trend of imported dengue cases in each country from overseas *Aedes*-borne disease epidemics [17].

On detecting autochthonous cases, the three countries implement some common response measures (site visits and blood donation safety protocols), yet differ in implementing others, including a heavy reliance on vector control in France and Italy and deployment of ad hoc entomological surveillance and xenomonitoring in Italy and Portugal. The exhaustiveness of and variations in response measures likely reflects different risk-tolerance for autochthonous transmission, the remit of entomological surveillance and associated allocated resources and infrastructure for its prevention, in each country.

France, Italy and Portugal systematically implement seasonal surveillance of invasive *Aedes* mosquitoes in both colonised areas and PoE, in line with the ECDC guidelines for the surveillance of invasive mosquitoes in Europe. In France and Portugal, coverage of the vector surveillance systems is national due to the increasing domestic colonisation of *Ae. albopictus* [18].

While Italy exhibits re-active molecular xenomonitoring (i.e. tied to the occurrence of human cases) Portugal employs pro-active xenomonitoring as early warning, implemented since the dengue outbreak in Madeira in 2012. Conversely, unlike France and Italy, Portugal does not yet monitor insecticide resistance due to low *Aedes* vector abundance.

Notably, all countries need to improve operational action in response to *Aedes* vector abundance fluctuations in colonised areas, which could impact corresponding surveillance activities. Conversely, all countries implement or foresee vector control interventions and ad hoc entomological surveillance activities in response to the detection of invasive *Aedes* species in non-colonised areas. This may prove crucial in preventing or reducing the extent of future colonisation events and, in the case of vector absence, in re-assessing local transmission risks. Moreover, some regional variation in vector surveillance and response measures exists within the countries, due to differences in vector colonisation, or regional autonomy and delegation of public and environmental health services. For example, a study in Italy found that municipalities located in lowlands, have longer infestation periods and possess greater economic resources are more prone to vector control than those that are more urbanised [19].

The epidemiology of *Aedes-*borne diseases is changing rapidly in Europe and we have illustrated how human and entomological surveillance and response systems are evolving in three countries experiencing different organisational, epidemiological and entomological drivers. We identified a range of good practices that could be applicable to a wider European perspective (Box).

Box **Good practices identified from Italy, France and Portugal surveillance systems, applicable to a wider European perspective**

• Active case finding through site visits in response to autochthonous cases ensures improved detection of unreported cases and reduce risk of ongoing transmission.

• Seasonal active surveillance during higher risk periods for arbovirus transmission improves early detection of clusters for response.

• Integrated analysis of epidemiological and entomological surveillance data provides better guidance to the risk assessment for response measures to autochthonous cases.

• Harnessing existing epidemic intelligence tools helps to improve early response.

• Insecticide resistance monitoring and xenomonitoring inform vector control and enable early warning about pathogen circulation.

• Cross-border sharing of good practices in surveillance enables harmonisation of detection, reporting and public health response.

If implemented, these practices can facilitate better comparison of national epidemiological and entomological indicators among countries and the establishment of shared baseline response practices against arboviral threats. Sharing may also assist countries setting up or upgrading their existing surveillance systems to more effectively respond to increasing colonisation by *Aedes* species and increasing *Aedes*-borne autochthonous cases. This in turn can facilitate appropriate risk-based tailoring of surveillance systems by comparative risk-based assessment of EU/EEA countries, to improve preparedness and early warning capacity at national level.

There is no one-size-fits-all, and we show how the surveillance strategy for *Aedes*-borne diseases and their vectors [7] is evolving in countries in response to drivers underlying the risk of local transmission. Benchmarking surveillance systems is essential to enable comparison of different systems, reflect on observed differences and provide policy options for countries that have not yet experienced local transmission of *Aedes*-borne diseases or an extensive colonisation by *Aedes* species. Overall, each country needs to consider its epidemiological and entomological situation, reflecting the level of risk as well as socioeconomic context, competing priorities, available resources and legal frameworks to identify the surveillance and response strategy that best meets its needs [20,21]. These aspects are also intertwined with the healthcare system centralisation/decentralisation level in each country, yielding varying surveillance and response trade-offs between, for example, localised decision-making and timely response, and national level coordination, and between regional level efficiency and resilience, and country wide system equity and quality performance. Moreover, having different surveillance systems in different EU/EEA countries, in terms of type, coverage and case definitions, may help detect cases/vectors that could go undetected in neighbouring EU/EEA countries. This diversity could, in turn, enhance the sensitivity and cost-effectiveness of surveillance systems throughout Europe, especially when local epidemiology and entomology does not support investing in similar surveillance systems.

Considering the cross-border nature of *Aedes*-borne arbovirus threats, disease burden interdependence between countries and increasing climatic suitability for *Aedes*-borne disease transmission due to climate change [22], establishing consensus of good practices by risk level may be the optimal approach to strengthen surveillance and improve preparedness, early warning and response for *Aedes*-borne diseases in Europe. Risk level may be informed by standardised risk-assessment of EU countries considering their epidemiological and entomological situation.

Our study has several limitations. Aiming to provide a comprehensive, national level overview of the surveillance and response to *Aedes*-borne diseases in each country, our analysis does not exhaustively account for all potential surveillance and response variations occurring within each country (e.g. by region or province). Furthermore, although we collated data from several sources (i.e. key-informant interviews, national guidelines and scientific literature) potential local level surveillance and response practices diverging from designated and/or after-action reported practice are not accounted for. Finally, while our analysis successfully integrated multiple benchmark objects related to human and entomological surveillance/response, future analyses can be further enriched by including additional important objects (e.g. the organisation of reference laboratory systems and differential diagnostic approaches, financial resources, and surveillance workforce).

## Conclusion

Our benchmarking analysis identified that France, Italy and Portugal currently operate broadly similar and evolving surveillance systems and response measures to *Aedes*-borne diseases and *Aedes* vectors. Variations appear to be dependent on three main drivers: (i) the healthcare system organisation, (ii) the extent of *Ae. albopictus* colonisation and (iii) the local transmission of *Ades-*borne diseases in each country.

The best-fit approach to *Aedes*-borne disease surveillance for a given country may be one of risk-based assessment based on epidemiological and entomological indicators in the country and in bordering and interconnected countries and territories. Implementation of harmonised, risk-based surveillance systems across Europe and enhanced cross-border collaboration can improve preparedness, control and response to *Aedes*-borne diseases.

## Supplementary Data

615000 Supplementary Material
