# Supplementary material for: Description and comparison of national surveillance systems and response measures for Aedes-borne diseases in France, Italy and Portugal: a benchmarking study, 2023
Source: Euro Surveill. 2025 Apr 17;30(15):2400515. doi: 10.2807/1560-7917.ES.2025.30.15.2400515 (PMC12007401; doi:10.2807/1560-7917.ES.2025.30.15.2400515)
Supplement: Supplementary Material [file 24-00515_LEITE_Supplementary_material.pdf]

Supplementary material

This supplementary material is hosted by *Eurosurveillance* as supporting information alongside the article 'Description and comparison of national surveillance systems and response measures for *Aedes*-borne diseases in three countries in France, Italy and Portugal: a benchmarking study, 2023', on behalf of the authors, who remain responsible for the accuracy and appropriateness of the content. The same standards for ethics, copyright, attributions and permissions as for the article apply. Supplements are not edited by *Eurosurveillance* and the journal is not responsible for the maintenance of any links or email addresses provided therein.

Supplementary Table S1. Questionnaire framework of benchmarking objects, criteria and description used to survey the human surveillance systems for Aedes-borne disease in France, Italy and Portugal (excluding overseas territories).

Benchmark Object	Benchmark criteria	Response type	Benchmark description
**Surveillance objectives**	defined	Y/N	the surveillance objectives are defined in a regional or national plan or document or legislation
**Diseases under surveillance**	arbovirus name	Y/N	disease is under surveillance
**Case definitions**	ECDC	Y/N	the case definition used is that of the ECDC https://eur-lex.europa.eu/legal-content/EN/TXT/PDF/?uri=CELEX:32018D0945&from=EN#page=18
	national	Y/N	the case definition used is other than that of the ECDC
	number of case definitions	number	number of case definitions
	suspected	free text	provide text of national case definition
	probable	free text	provide text of national case definition
	confirmed	free text	provide text of national case definition
**National surveillance data flow**	schema	free text	schema data of the data flow and co-ordination
	clinicians	Y/N	notification/reporting of cases is received from community doctors
	hospitals	Y/N	notification/reporting of cases is received from hospital doctors
	laboratories	Y/N	notification/reporting of cases is received directly from laboratories
	feedback mechanism	Y/N	are there feedback mechanisms for data flow
**Population under surveillance**	General population	Y/N	surveillance of the general population
	Risk groups	free text	specify the specific risk groups (blood donors, organ transplanters…) under routine surveillance
**Geographic coverage**	National	Y/N	the surveillance system covers all regions of the country
	Regional	Y/N	the surveillance system covers certain regions
	Regional coverage	number	numerator (covered regions) / denominator (total number of regions)
	Regional variations	Y/N	there are variations in the surveillance coverage between regions
**Type of surveillance:**	Passive	Y//N	the clinician/hospital/laboratory takes the initiative to report the case)
	Active	Y/N	active (the public health agency takes the initiative to check for cases)
	Compulsory	Y/N	compulsory (reporting of cases is mandatory under legislation)
	Voluntary	Y/N	voluntary (reporting of cases is voluntary)
	Comprehensive	Y/N	comprehensive (all cases within the area covered by the surveillance system are reported)
	sentinel	Y/N	sentinel (only certain selected sites within the area report cases)
	Perennial	Y/N	perennial (the surveillance system runs throughout the entire year)

	Seasonal	Y/N	seasonal (the surveillance system runs only during peak/high risk months)
	Syndromic surveillance	Y/N	a set of clinical criteria alone are used to routinely report possible cases (e.g. fever + myalgia + rash)
	Event based	Y/N	non-standardized information collection, screening, assessment and interpretation
	Risk-based surveillance	Y/N	the extent, duration, and coverage of surveillance is based on risk assessment of high/low incidence
	Participative surveillance	Y/N	surveillance data are reported from field actors on a voluntary basis (general public)
	Early warning component	Y/N	detection of early warning and alerts
**Legal framework**	regulatory legistaltion	Y/N	the disease is notifiable under national law.
	Data linkage between datasets	Y/N	legal framework to link datasets from different data sources (e.g. laboratory data, vaccination data, mortality data…)
**Specification of information**	Case-based	Y/N	individual cases are reported
	Aggregated-based	Y/N	clusters or groups of cases are reported without individual case detail
	Variable specification	free text	list the mandatory variables reported (e.g.. symptom onset date, testing date, travel history, age, sex, hospitalisation)
	Frequency of data collection	daily / weekly / monthly	cases are notified daily / weekly / monthly to the public health agency
	Frequency of data reporting	daily / weekly / monthly	the public health agency reports cases to the national focal point or IHR daily / weekly / monthly
**Reporting format**	paper-based	Y/N	reporting and collection of data is paper-based or involves transcription from paper to electronic format
	electronic	Y/N	reporting and collection of data is conducted electronically
**Data entry**	web-based	Y/N	manual input of data into web-based portal or page
	Interface-mediated data entry	Y/N	data is exported automatically from one database (e.gh. Lab, hospital) to another (e.g. public health agency)
	Open-source software	Y/N	open-source software used to upload data
**Database architecture**	Centralised	Y/N	database based nationally, systems are able to run all necessary checks for data quality monitoring.
	Regionalized database	Y/N	database based regionally
	Data linkage between datasets	Y/N	platform in use for data linkage between datasets from different data sources (e.g. laboratory data, vaccination data, mortality data, entomological data…)
	Server selection	free text	type of software and hardware used to store surveillance data
**Alert threshold**	alert threshold to response	Y/N	a defined indicator level or threshold exists which prompts to response
	alert thresholds definition	free text	definition of the thresholds applied to respond
**Response actions**	site visit	free text	field visit to conduct further epidemiological surveillance (for imported vs local case, for single vs cluster of cases)
	active case-finding	free text	surveys of household contacts or door-to-door investigation to check for clinical criteria of probable cases (for imported vs local case, for single vs cluster cases)
	free testing	free text	free access to testing and healthcare to exposed not covered by the National Health System (for imported vs local, for single vs cluster)
	seroprevalence survey	free text	sampling of outbreak or local population with POCT or phlebotomy to assess for previous (IgG) or recent exposure (IgM) (for imported vs local case, for single vs cluster cases)
	communication to general public	free text	public awareness (for imported vs local case, for single vs cluster cases)
	national communication	free text	communication to health professionals(for imported vs local case, for single vs cluster cases)
	international communication	free text	communication to Member States, ECDC, WHO or others(for imported vs local case, for single vs cluster cases)
	blood transfusion safety measures	free text	screening is initiated for blood transfusion processes (for imported vs local, for single vs cluster)

organ transplantation safety measures	free text	screening is initiated for organ transplantation processes (for imported vs local, for single vs cluster)
surveillance of risk groups	free text	specify the specific risk groups(blood donors, organ transplanters, pregnant women...)
Adulticiding	Y/N	adulticide applications are performed in response to cases
Larviciding	Y/N	larvicide applications are performed in response to cases
Defined perimeter/number of insecticide applications	Y/N	perimeter (meters) of intervention around case residence, work area etc, and number of interventions
Environmental management	Y/N	environmental interventions for the destruction of natural or man-made breeding sites/resting sites is performed in response to cases
Molecular xenomonitorin	Y/N	Detection and identification of arboviruses in vector populations in response to case reporting
Ad hoc vector surveillance	Y/N	ad hoc entomological surveillance activities in response to case(s) reported

Supplementary Table S2. Questionnaire framework of benchmarking objects, criteria and description used to survey the human surveillance systems for Aedes invasive mosquitoes in France, Italy and Portugal (excluding overseas territories).

Benchmark Object	Benchmark Criteria	Response type	Benchmark description
**Surveillance objectives**	defined	Y/N	the surveillance objectives are defined in a regional or national plan or document or legislation
**Vector surveillance data flow**	schema	*free text*	schema data of the data flow and co-ordination
	environmental agencies	Y/N	environmental agencies involved in data collection and/or reporting
	private companies	Y/N	private companies involved in data collection and/or reporting
	citizen reporting	Y/N	notification/reporting of entomological data by citizens/citizen apps
	feedback mechanism	Y/N	are there feedback mechanisms for data flow
**Geographic coverage**			
	National	Y/N	the surveillance system covers all regions of the country
	Regional	Y/N	the surveillance system covers certain regions
	Points of entry	*free text*	active surveillance of possible introductions at PoE (points of entry)
	Colonised areas	*free text*	active surveillance of colonised areas
**Type of surveillance:**	Active	Y/N	active mosquito collection (e.g. by trapping adults, dipping preimaginal developmental stages or ovitrapping)
	Passive	Y/N	passive data collection (recognition, reporting, collection and submission of mosquito specimens by citizens)
	Systematic	Y/N	surveillance is conducted systematically at the spatial and temporal levels between and within seasons
	participative surveillance	Y/N	surveillance data are reported from field actors on a voluntary basis (general public)
	risk-based surveillance	Y/N	the extent, duration, and coverage of surveillance is based on risk assessment of high/low incidence
	voluntary	Y/N	reporting of indicators is voluntary
	compulsory	Y/N	reporting of indicators is compulsory
	event-based	Y/N	non-standardized information collection, screening, assessment and interpretation
	seasonal	Y/N	perennial (the surveillance system runs throughout the entire year)
	perennial	Y/N	seasonal (the surveillance system runs only during peak/high risk months)
**Reporting format**	paper-based	Y/N	reporting and collection of data is paper-based or involves transcription from paper to electronic format
	electronic	Y/N	reporting and collection of data is conducted electronically
**Data entry**	web-based	Y/N	manual input of data into web-based portal or page
	Interface-mediated data entry	Y/N	data is exported automatically from one database (e.gh. Lab, hospital) to another (e.g. public health agency)
	Open-source software	Y/N	open-source software used to upload data
**Specification of information to be reported**	presence absence	Y/N	reporting of vector presence or absence
	Abundance and seasonal dynamics	Y/N	reporting of vector abundance and seasonal dynamics
	molecular xenomonitoring	Y/N	entomological samples screened for arboviruses
	insecticide resistance monitoring	Y/N	insecticide resistance monitoring in vector specimens
	Frequency of data collection	Y/N	data are notified daily / weekly / monthly to the public health agency
	Frequency of data reporting	Y/N	the public health agency reports data to the national focal point or IHR daily / weekly / monthly
	mapping strategy	free text	vector distribution at level of country, regional, province, municipality
**Response actions**	Ad hoc surveillance	free text	ad hoc entomological surveillance activities preformed in response to routine entomological surveillance findings (e.g. in response to the detection of vectors in new geographical areas)
	Vector control	free text	Adulticiding, larviciding, environmental management or other actions performed to control vector populations (e.g. in context of high density of vectors or introduction of invasive species in previously non colonised area)
	Xenomonitoring	free text	Detection and identification of pathogens in vector populations
	communication to general public	free text	public awareness (vector presence, vector control, personal preventive measures)
	national communication	free text	communication to stakeholders (vector presence, dynamics, etc.)

international communication	free text	communication to Member States, ECDC, WHO, EFSA or others(vector presence, dynamics, etc.)

Supplementary Table S3. Routine human surveillance systems and response measures to Aedes-borne disease in Italy, France and Portugal (excluding overseas territories) as of 2023; summary table of results.

Benchmark	Benchmark criteria	France	Italy	Portugal
**Case definitions**	Compatible with ECDC	Y	Y	Y
	Case classificaiton	possible, probable, confirmed	possible, probable, confirmed	– probable, confirmed
**Population under routine surveillance**	General population	Y	Y	Y
	Risk groups	N	N	N
**Geographic coverage**	National	Y	Y	Y
	Regional variations	N	N	N
**Surveillance type**	Passive	Y	Y	Y
	Active	Y	N	N
	Compulsory	Y	Y	Y
	Comprehensive	Y	Y	Y
	Perennial	Y	Y	Y
	Seasonal	Y	N	N
	Event based	N	Y	Y
	Risk-based surveillance	Y[1]	Y[1]	N
	Participative surveillance	N	N	N
	Early warning component	Y	Y	Y
**Information specification**	Case-based	Y	Y	Y
	Frequency of data collection	daily	daily	daily
	Frequency of data reporting	daily (national) / annual (ECDC)	daily (national) / annual (ECDC)	daily (national)/ (international)
**Reporting format**	Paper-based	Y	N	N
	Electronic	Y	Y	Y
**Database architecture**	Manual web-based entry	Y	Y	Y
	Centralised / national	Y	Y	Y
	Database linkage	Y	Y	Y
**Alert threshold**	Alert threshold for response	1 confirmed autochthonous case	1 autochthonous probable or confirmed case	1 case
**Response measures to human cases**	Site visit	Y	Y	Y
	Active case-finding	Y	Y	Y
	Free testing	Y	Y	Y
	Seroprevalence survey	N[2]	Y[2]	N
	General public communication	Y	Y	Y
	National communication	Y	Y	Y
	International communication	Y	Y	Y
	Blood transfusion safety measures	Y	Y	Y
	Organ transplantation safety measures	N	Y	Y
	Surveillance of risk groups	blood donors	blood and organ transplant donors	blood and organ transplant donors

**Vector related response measures to human cases**	Adulticiding	Y	Y	N
	Larviciding	Y	Y	N
	Defined perimeter/number of insecticide applications	Y	Y	N
	Environmental management	Y	Y	N
	Molecular xenomonitoring	N	Y	Y[3]
	Ad hoc vector surveillance	N	Y	Y[3]

[1] France employs active surveillance, during the high-risk season where retrospective checking of laboratory result databases is performed to actively search for unreported cases. Italy carries out clinician awareness campaigns and enhanced reporting/information dissemination at the national level during the high-risk season.

[2]Seroprevalence surveys, are not routinely conducted in France; are foreseen in Italy and were conducted in the majority of recent dengue outbreaks. [3] Ad hoc molecular xenomonitoring and ad hoc surveillance activities are planned in Portugal.

Supplementary Table S4. Routine vector surveillance systems and response measures to *Aedes* species in Italy, France and Portugal (excluding overseas territories) as of 2023; summary table of results.

Benchmark	Benchmark criteria	France	Italy	Portugal
**Vector surveillance data flow**	Stakeholders involved	Local environmental health agency, Health Ministry, National environmental health	Local Public Health authority, Regional Public Health authority	Local public health authority, National reference laboratory
	Environmental agencies	Y	N	N
	Private companies	Y	Y	Y
	Citizen reporting	Y	Y	Y
	Feedback mechanism	Y	Y	Y
**Geographic coverage**	National	Y	N[1]	Y
	Regional variations	Y	Y	N
	Points of entry	Y	Y	Y
	Colonised areas	Y	Y	Y
**Surveillance type**	Active	Y	Y	Y
	Passive	Y	Y	Y
	Systematic	Y	Y	Y
	Participative surveillance	Y	Y[2]	Y
	Risk-based surveillance	Y	Y	Y
	Voluntary	N	N	N
	Compulsary	Y	Y	Y
	Event-based	Y	Y	Y
	Seasonal	Y	Y	Y
**Database architecture**	Manual web-based entry	Y	Y	Y
	Interface-mediated	Y	N	N
	Open-source software	N	N	Y
**Specification of information**	Presence / absence	Y	Y	Y
	Abundance and seasonal dynamics	Y	Y	Y
	Molecular xenomonitoring	N	N	Y
	Insecticide resistance monitoring	Y[3]	Y[3]	N
	Frequency of specimen collection	bimonthly	bimonthly	weekly
	Frequency of data reporting	flexible	na	flexible
	Mapping strategy	municipality	municipality	local health authority
**Alert threshold**	Alert threshold for response	detection of invasive Aedes species in previous non-colonised municipality	detection of invasive Aedes species in previous non-colonised municipality	detection of invasive Aedes species in previous non-colonised municipality
**Response measures to routine entomological surveillance** (independent of human cases)	Vector control	Y[4]	Y[4]	Y
	Adulticiding	Y[4]	Y[4]	N
	Larviciding	Y[4]	Y[4]	Y
	Environmental management	Y[4]	Y[4]	Y

	Ad hoc entomological surveillance	Y	Y	Y
	Xenomonitoring	N	N	Y
	General public communication	Y[5]	Y[5]	Y[5]
	National communication	Y[5]	Y[5]	Y[5]
	International communication	Y	Y	Y

[1]In Italy Aedes vector surveillance coverage is regional, future implementation of a national coverage system is planned.

[2]In Italy participative surveillance is currently implemented as part of pilot/proof of concepts experiences

[3]Resistance monitoring is conducted periodically in France and systematically in Emilia-Romagna and Veneto regions in Italy.

[4]Vector control measures are foreseen upon invasive Aedes specimen detection in previously non-colonized areas. However in the absence of autochthonous transmission, vector control measures are not routinely performed in known colonised areas.

[5]Communications include the detection of invasive species in previously non colonised areas but also vector abundance fluctuations in previously/known colonised areas.

Supplementary Figure S1.

A Schema of information flow for human surveillance of *Aedes*-borne disease in France (excluding overseas territories)

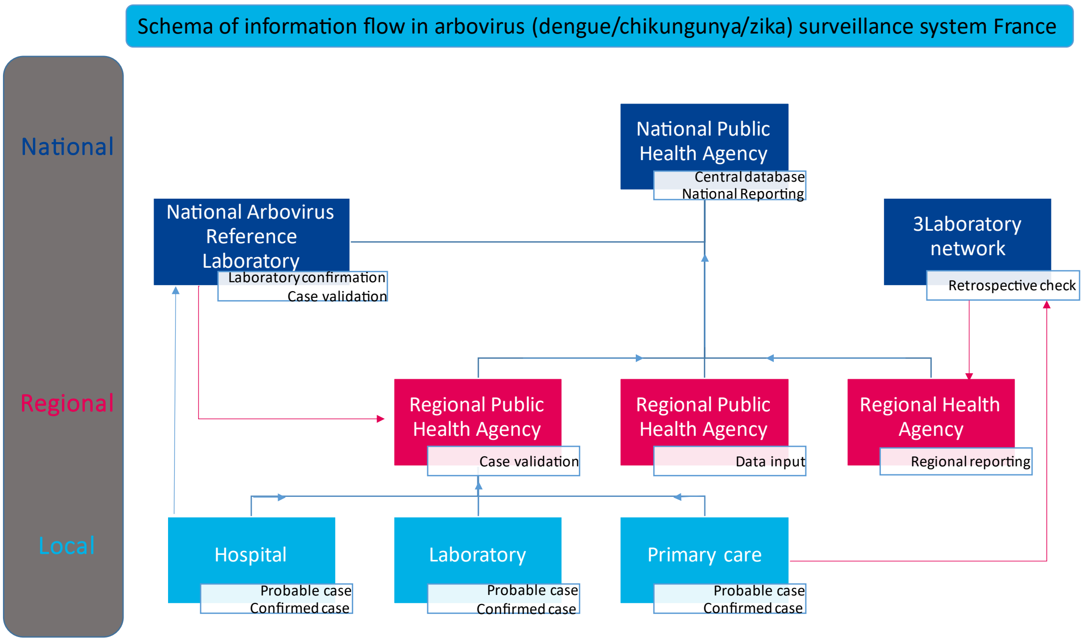

B Schema of information flow for human surveillance of *Aedes*-borne disease in Italy

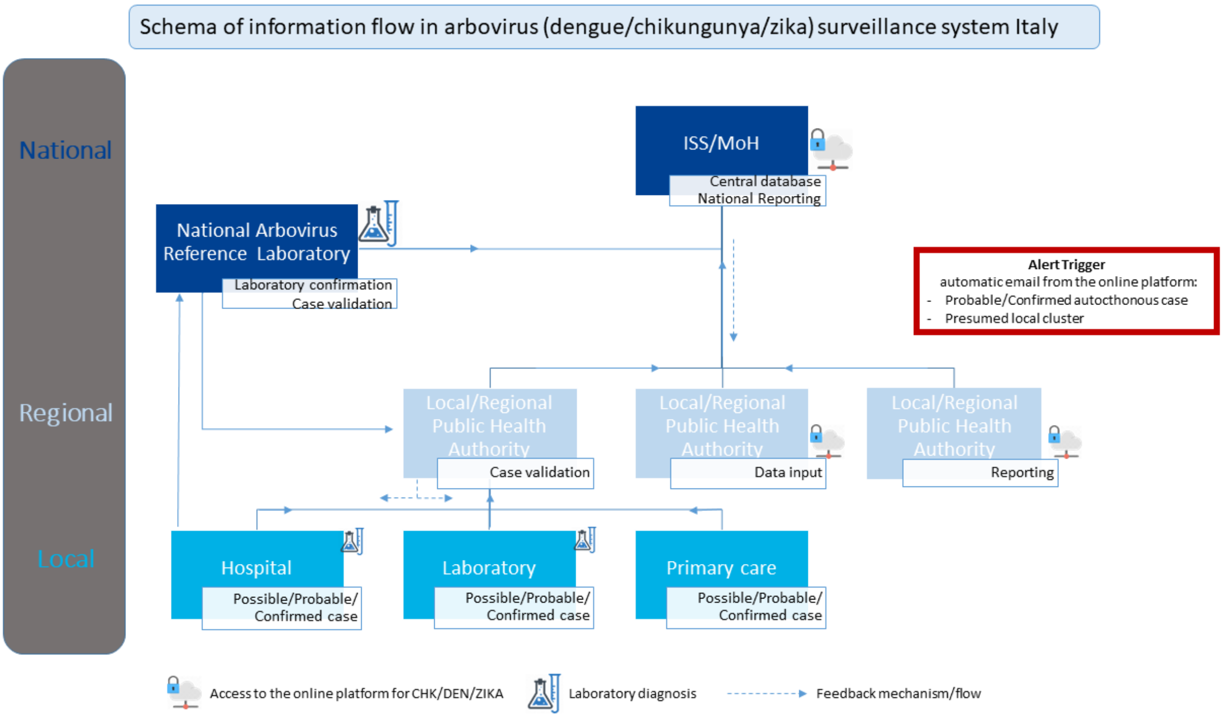

C Schema of information flow for human surveillance of *Aedes*-borne disease in Portugal

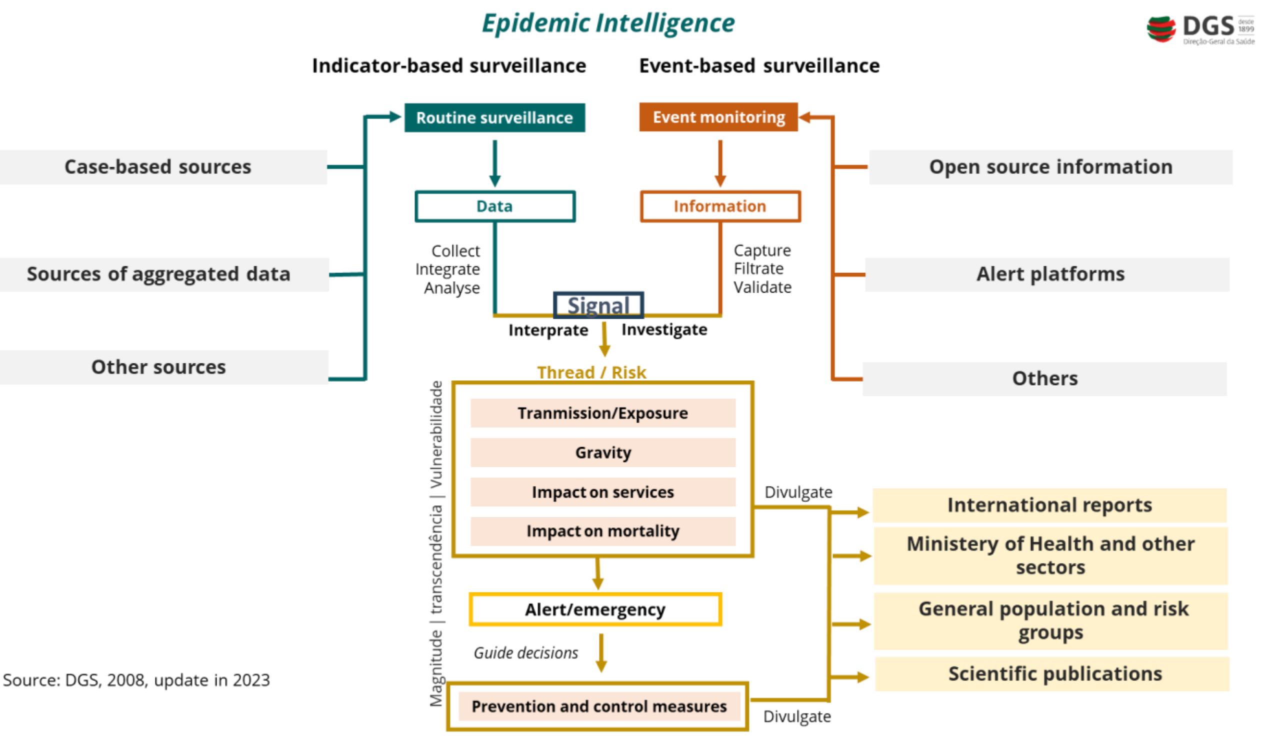

Source: DGS, 2008, update in 2023
